# Strut and radio-morphometric analysis of mandibular trabecular structure in pre-and post-menopausal women to aid in the diagnosis of osteoporosis

**DOI:** 10.1016/j.jobcr.2024.03.002

**Published:** 2024-03-22

**Authors:** Ragavendiran Anandan, Krithika C.L, Anuradha Ganesan, Yesoda Aniyan K.

**Affiliations:** Department of Oral Medicine and Radiology, SRM Dental College, no.1 Bharathi Salai, Ramapuram, Chennai-600089, India

**Keywords:** Osteoporosis, Radio-morphometric indices, Image texture, Strut analysis, Orthopantomogram

## Abstract

**Purpose:**

The purpose of the study is to evaluate the mandibular trabecular pattern in pre- and postmenopausal age women. By analysing the strut, fractal, grey level co-occurrence matrix, and radio-morphometric indices in the panoramic radiograph.

**Method:**

Panoramic radiographs from 2019 to 2022 were used to assess pre- and postmenopausal women's bone mineral density. A total of 272 panoramic radiographs, which exhibited clear visibility of the mental foramen on both sides without any blurring, motion artefacts, surgical errors, overlapping hyoid bone, or inferior mandibular cortex, were divided into two groups. Group A (136 premenopausal women) and Group B (136 postmenopausal women). It is a retrospective study that is non-interventional/observational in design. Strut features, fractal dimensions, a grey-level co-occurrence matrix, and radio morphometric indices were used to investigate bone texture in an image processing program. The mean difference between group variables was calculated using an independent sample *t*-test/unpaired *t*-test.

**Results:**

Pre-menopausal women had a mean age of 38.83 ± 6.01 years, while postmenopausal women had a mean age of 68.26 ± 8.31 In the postmenopausal group Four regions of interest exhibited fractal dimensions with a P value of less than 0.01 and GLCM features including contrast (0.812), correlation (0.230), energy (0.215), and homogeneity (0.322). Strut features of the four regions showed that 15 of 19 characteristics were significantly different.

**Conclusion:**

Orthopantomogram is useful in screening for osteoporosis. Strut, radio-morphometric indices, and fractal analysis can assess bone texture and quality. Future research incorporating artificial intelligence can revolutionize image analysis and support clinical decision-making.

## Graphical abstract

**Figure:**
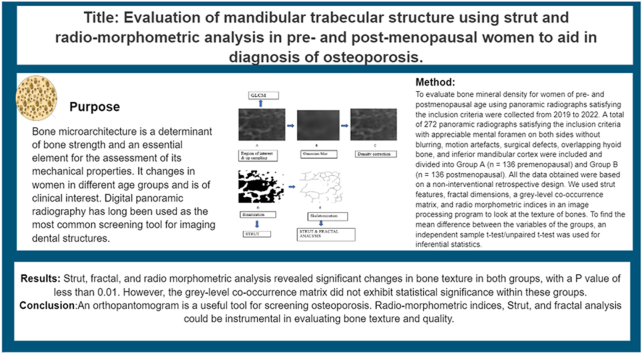

## 1 Introduction

According to the World Health Organisation (WHO), natural menopause is defined as at least 12 months of amenorrhoea that are not due to pathological or physiological factors. As per statistics, the average age of natural menopause is 51 years in developed countries, whereas it is 48 years in developing and underdeveloped countries. Given that the average lifespan has been increased to 70 years, the majority of women will live longer than one-third of their lives after going through menopause. In addition, as the ageing population grows faster than ever, the percentage of menopausal women is increasing. As a result, menopausal women's health is now a major global concern.^1^ Osteoporosis is a widely recognised and prevalent condition affecting the entire skeletal system. It is marked by reduced bone density and degeneration of the bone structure, leading to increased vulnerability to fractures.^2^, ^3^, ^4^ Early osteoporosis diagnosis could lower the risk of fracture and enhance the quality of life, especially in postmenopausal women. Panoramic radiographs are now commonly recommended for patient education and to gather helpful data about obtaining various forms of general dental care.^5^ Numerous studies have also found strong associations between the bone mineral density (BMD) of the mandible and the osteoporosis prevalence in the lumbar spine, femoral neck, and forearm.^6^

Hormones have a significant effect on bone health in women. Oestrogen is crucial in maintaining bone mineral density in women at various stages of life. A decrease in oestrogen levels can significantly affect bone mineral density, attributing to the increased risk of osteoporosis development in postmenopausal women.^7^ Various other hormones, like testosterone, parathyroid hormone (PTH), calcitonin, and growth hormone, also regulate the processes of bone formation and resorption.^8^

The utilization of objective mathematical analysis, such as fractal dimension (FD) and the grey level co-occurrence matrix (GLCM), have become more important when conducting an investigation. Nevertheless, research on these techniques has yielded contradictory findings, primarily due to insufficient emphasis on the selection of a specific region of interest and the fine-tuning of parameters to achieve optimal binarization.^9^ Quantitative morphologic methods like strut analysis have been extensively employed in the medical field, including trabecular pattern analysis, to quantify the structural components of various objects. Radio-morphometric indices provide insight into the morphological aspects of bone.^10^

Orthopantomogram is a cost-effective tool that is often indicated for evidence-based, comprehensive oral diagnosis. These radiographs can be used by patients in the susceptible age group to screen for osteoporosis by using various inexpensive software systems. Through a literature search, it has been found that OPG has the potential to detect osteoporosis in a manner that is comparable to the gold standard technique. Only a limited number of studies have assessed these factors in two separate cohorts of women. The objective of this study is to analyze changes in the microarchitecture of bone by evaluating alterations in bone texture using strut analysis, fractal dimensions, GLCM, and radio-morphometric indices. The study also aims to compare these features between pre- and post-menopausal individuals and identify specific deviations that may indicate bone health.

## 2 Materials and methods

### 2.1 Study design and participants

A total of two seventy-two panoramic radiographs, acquired at our dental institution from 2019 to 2022, were selected for the study based on the inclusion criteria. The Institutional Review Board (IRB) approval number for the ethical statement was: SRMU/M&HS/SRMDC/2022/PG/002. All the data collected followed a retrospective study that is non-interventional/observational in design and were analysed anonymously. The sample size was determined by using an effect size of 0.4, an alpha error of 0.05, and a power of 0.95. The overall sample size was calculated to be 272, with 136 participants allocated to each group. Criteria for inclusion: The study comprised female patients between the ages of 25 and 40 in group A. (n = 136). In group B female patients between the ages of 50 and 70 (n = 136). The investigation comprised radiographs that clearly showed the mental foramen on both sides, without any blurring, motion artefacts, surgical flaws, overlapping hyoid bone, or inferior mandibular cortex. Radiographs exhibiting intrinsic faults and patient positioning errors were eliminated. The trained oral and maxillofacial radiologist selected the images that met the inclusion criteria using a calibrated 21.3-inch colour monitor. Using strut, fractal, GLCM, and radio-morphometric analysis on panoramic X-rays to look at the mandible's trabecular pattern was the main goal. The secondary objective was to compare different indices of bone mineral density assessment in women of reproductive age and postmenopausal women.

### 2.2 Procedures

**Fig. 1:**
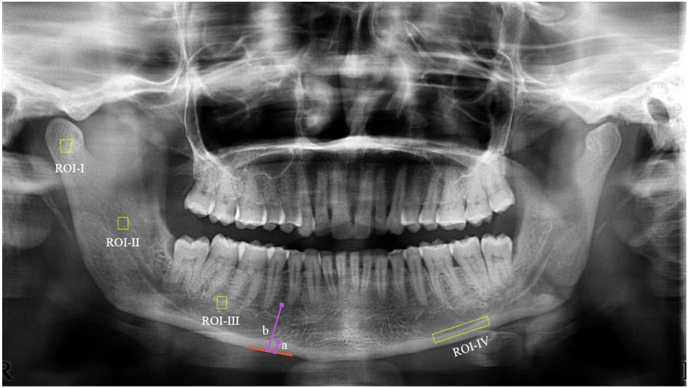
Selection of the region of interest. ROI-1 represents the central area of the condylar head without any degenerative conditions. ROI-2 corresponds to the central area of the ramus region. ROI-3 without periapical radiolucency or sclerosis below and between the molars. If a molar was absent, the middle area horizontally 2 cm medial from the oblique line-ramus intersection were chosen. ROI-4: The endosteal margin. Measurement of mental index line b measures mandibular cortical thickness at the mental foramen midway, perpendicular to the lower edge of the mandible. Measurement of Panoramic mandibular index line a measure mandibular cortical thickness at the mental foramen midway, perpendicular to the lower edge of the mandible.

**Selection of Region of Interest:** An individual observer selected four regions of interest (ROI) from all of the images was chosen bilaterally with a few overlapping structures and noise. based on Hwang et al.^10^ (ROIs) were selected within the medullary area, as shown in (Fig. 1). The ROIs were defined with a fixed square dimension of 5 × 5mm. ROI-1 represents the central area of the condylar head without any degenerative conditions. ROI-2 corresponds to the central area of the ramus region. ROI-3 without periapical radiolucency or sclerosis below and between the molars. If a molar was absent, the middle area horizontally 2 cm medial from the oblique line-ramus intersection was chosen. ROI-4: The endosteal margin (Fig. 1).

**Analytical software:** The Image J software was used to select regions of interest (ROIs) and perform intermediate processing. The feature analysis was conducted using MATLAB. We generated a customized computer application using MIJ version 1.3.9, which is Java software designed specifically for exchanging images between MATLAB and ImageJ (version 1.6). This software was developed by the Biomedical Imaging Group. During the process of exchanging images between MATLAB and ImageJ, the integrity of the image is preserved by conducting a thorough analysis of pixel values and statistical properties to ensure the accuracy of the exchanged images.

**Fig. 2:**
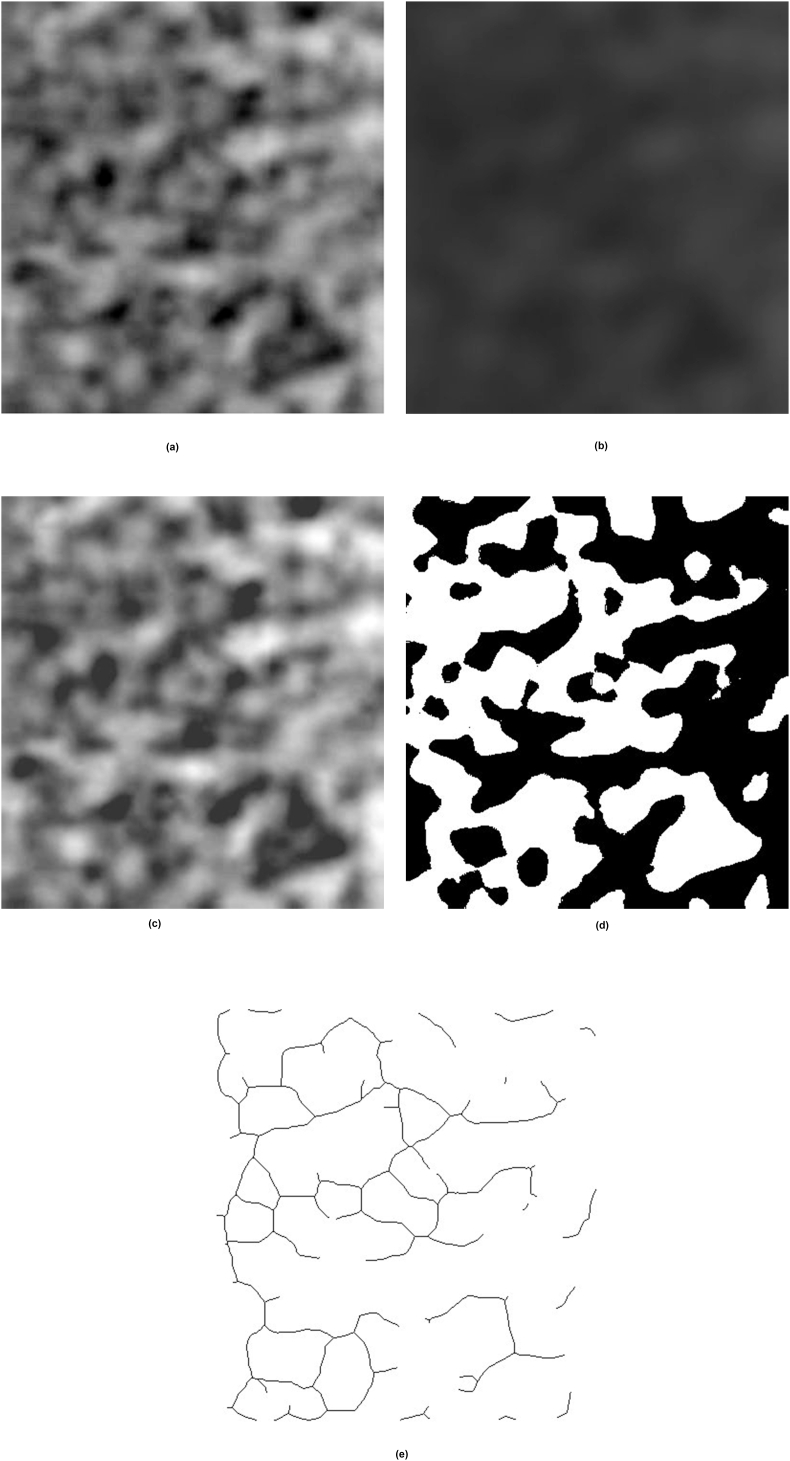
Shows image processing 2(a)ROI selection and up sampling, 2(b) Gaussian blur, 2(c) Density correction,2(d) Binarization, 2(e) Skeletonization.

**Image processing technique for analysis:** The regions of interest (ROIs) were identified, and subsequently, the grey-level co-occurrence matrix (GLCM) was calculated. The image was processed using the White and Rudolph method.^11^ It was enlarged by 400% using bicubic interpolation with a sigma of 35 and a filter size of 33(Fig. 2a). A Gaussian filter was applied to blur the image (Fig. 2b). The density adjustment was done by subtracting the blurred image from the original (Fig. 2c). After adding a grey value of 128 to each pixel, the binarization (Fig. 2d) and skeletonization (Fig. 2e) operations were performed. The binary images were utilised to conduct strut and fractal analysis. The radio-morphometric indices were assessed using identical pictures.

**Grey Level Co-occurrence Matrix (GLCM):** The GLCM was employed to examine textural characteristics by utilising a second-order statistical method that characterises the different shades of grey present in an image. For each region of interest (ROI), this study examined and analysed the measures of contrast, correlation, energy, and homogeneity.

**Fractal dimensions analysis:** The fractal dimension was utilised to create a statistical indicator for comparing the complexity of changes in the trabecular morphological pattern with a measurement of scale. The skeletonized pictures acquired from image processing were analysed using the box-counting approach.

**Strut analysis**: Analysed using a binary image, the high-density zone and the length of the periphery were determined. White pixels served as a marker for the high-density area. The peripheral refers to the outer boundary of the region with high density. The binary image was skeletonized to examine the structural components, including nodes (crossing points), termini (free ends), and struts (connections between two other parts), and the characteristics evaluated are HDA (High-Density Area) are N (Number) and Nd (Nodes). The perimeter of the HAD corresponds to the total number of pixels at its outer boundary. Tm refers to the endpoints, whereas TSL stands for the overall length of the struts. To facilitate comparisons, each attribute was expressed as a ratio of its related length, area, or perimeter. (Table 1).

**Table 1:** Comparison of mean difference of four Roi among pre-menopausal and post-menopausal age group.

VARIABLES	ROI-1					ROI-2					ROI-3					ROI-4				
	PRE-MENOPAUSAL		POST-MENOPAUSAL		P-VALUE	PRE-MENOPAUSAL		POST-MENOPAUSAL		P-VALUE	PRE-MENOPAUSAL		POST-MENOPAUSAL		P-VALUE	PRE-MENOPAUSAL		POST-MENOPAUSAL		P-VALUE
	MEAN	SD	MEAN	SD		MEAN	SD	MEAN	SD		MEAN	SD	MEAN	SD		MEAN	SD	MEAN	SD	
**FRACTAL DIMENSIONS**	1.38	0.07	1.32	0.04	<0.01*	1.2	0.03	1.2	0.08	0.035*	1.2	0.11	1.2	0.11	<0.01*	1.2	0.11	1.2	0.11	<0.01*
**GLCM**																				
**CONTRAST**	0.22	0.01	0.22	0.01	0.042*	0.24	0.04	0.23	0.03	0.467	0.46	0.02	0.44	0.03	0.812	0.46	0.02	0.44	0.03	0.812
**CORRELATION**	0.91	0.05	0.90	0.03	0.131	0.92	0.01	0.92	0.01	0.663	0.92	0.04	0.92	0.02	0.230	0.92	0.04	0.92	0.02	0.230
**ENERGY**	0.11	0.01	0.10	0.003	0.955	0.16	0.01	0.14	0.01	0.073	0.12	0.01	0.12	0.006	0.215	0.12	0.01	0.12	0.006	0.215
**HOMOGENEITY**	0.77	0.05	0.72	0.03	0.916	0.86	0.04	0.82	0.05	0.395	0.86	0.03	0.84	0.03	0.322	0.86	0.03	0.84	0.03	0.322
**STRUT**																				
**HDA/TOTAL AREA**	0.48	0.01	0.47	0.01	<0.01*	0.48	0.02	0.46	0.03	<0.01*	0.46	0.01	0.48	0.008	0.007*	0.48	0.01	0.50	0.008	0.007*
**PERIPHERY/TOTAL AREA**	0.021	0.004	0.01	0.006	0.061	0.01	0.007	0.01	0.001	<0.01*	0.01	0.003	0.01	0.004	0.066	0.01	0.003	0.01	0.004	0.071
**PERIPHERY/HDA**	0.016	0.01	0.01	0.007	<0.01*	0.019	0.002	0.01	0.001	<0.01*	0.02	0.004	0.02	0.002	<0.01*	0.02	0.004	0.02	0.002	<0.01*
**TSL/HAD**	0.024	0.006	0.02	0.008	0.063	0.25	0.05	0.22	0.02	<0.01*	0.02	0.008	0.02	0.003	0.001*	0.02	0.007	0.02	0.004	0.001*
**TSL/TOTAL AREA**	0.012	0.003	0.01	0.001	<0.01*	0.01	0.003	0.008	0.002	0.01*	0.01	0.006	0.008	0.001	<0.01*	0.01	0.005	0.008	0.001	<0.01*
**N.TM/SQ CM**	0.07	0.007	0.01	0.003	<0.01*	0.08	0.009	0.07	0.007	<0.01*	0.68	0.05	0.65	0.03	<0.01*	0.68	0.05	0.68	0.03	<0.01*
**N.TM/TSL**	7.64	0.12	7.6	0.05	<0.01*	7.45	0.40	7.55	0.25	0.011*	2.27	0.067	2.24	0.05	<0.01*	2.27	0.067	2.21	0.05	<0.01*
**N.TM/PERIPHERY**	4.18	0.03	4.16	0.02	<0.01*	4.12	0.03	4.08	0.02	<0.01*	1.18	0.01	1.13	0.013	<0.01*	1.15	0.01	1.11	0.019	<0.01*
**N.TM/HAD**	0.15	0.004	0.14	0.01	<0.01*	0.01	0.003	0.01	0.002	<0.01*	0.14	0.02	0.13	0.01	<0.01*	0.16	0.02	0.13	0.01	<0.01*
**N. ND/SQ CM**	0.05	0.004	0.04	0.003	<0.01*	0.008	0.002	0.06	0.001	<0.01*	0.007	0.03	0.005	0.00	<0.01*	0.007	0.03	0.005	0.00	<0.01*
**N. ND/TSL**	4.91	0.03	4.8	0.04	<0.01*	4.83	0.04	4.82	0.03	0.114	5.05	0.019	5.04	0.02	0.021*	5.05	0.018	5.02	0.03	0.021*
**N. ND/PERIPHERY**	2.74	0.07	2.68	0.01	<0.01*	2.74	0.08	2.73	0.06	0.198	1.74	0.46	1.55	0.02	<0.01*	1.74	0.46	1.52	0.02	<0.01*
**N. ND/HAD**	0.10	0.01	0.09	0.10	<0.01*	0.10	0.008	0.10	0.002	<0.01*	0.02	0.006	0.02	0.003	0.018*	0.02	0.006	0.02	0.03	<0.01*
**N. ND/N.TM**	0.52	0.01	0.051	0.001	<0.01*	0.63	0.042	0.62	0.03	0.073	0.46	0.02	0.44	0.02	<0.01*	0.46	0.02	0.44	0.02	<0.01*

GLCM- Grey level co-occurrence matrix; HDA, or High-Density Area; N, or Number; Nd, or Nodes; The perimeter of the HAD refers to the overall count of pixels along its outer margin. Tm denotes the termini, while TSL represents the total length of the struts.

**Fig. 3:**
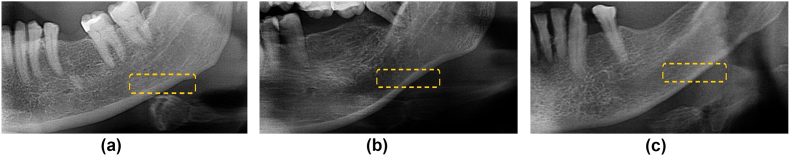
Klemetti classification,3(a) C1 represents a cortex that is within the normal range, 3(b) C2 indicates a cortex that is mild to moderately affected,3(c) C3 represents a cortex that is severely eroded.

**Mandibular Cortical Index (MCI):** The MCI was determined using the Klemetti classification, which assesses the visibility of the cortical border of the mandible beyond the mental foramina. C1 represents a cortex that is within the normal range(Fig. 3a), C2 indicates a cortex that is mild to moderately affected (Fig. 3b), and C3 represents a cortex that is severely eroded, (Fig. 3c).

**Mental index (MI):** The mental index (MI) was assessed (Fig. 1) by measuring the thickness of the mandibular cortex at the midpoint of the mental foramen, perpendicular to the lower edge of the mandible marked as b in (Fig. 1) (normal value > 3.1 mm).

**Panoramic Mandibular Index (PMI):** It is a diagnostic tool used to assess the bone mineral density of the mandible. The PMI is determined by dividing the mandibular cortical thickness, measured perpendicularly to the bottom of the mandible at the midpoint of the mental foramen, by the distance between the upper edge of the lower mandibular cortex and the bottom of the mandible, marked as a in (Fig. 1) A normal PMI value is greater than 0.3.

### 2.3 Statistical analysis

The analysis followed a normal distribution, according to the results of the normality, Kolmogorov-Smirnov, and Shapiro-Wilks tests. Thus, a parametric test was used to analyze the data. The mean and standard deviation were used to express descriptive statistics. Inferential statistics was used to determine the mean difference between the variables in the pre and postmenopausal groups using an independent sample *t*-test/unpaired *t*-test. SPSS (IBM SPSS Statistics for Windows, Version 26.0, Armonk, NY: IBM Corp. Released 2019) have been used to analyze the data. The level of significance was set at 5% (=0.05). Statistical significance was defined as a P-value of 0.05 or lower.

## 3 Results

The mean age of pre-menopausal women was 38.83 ± 6.01 years while the mean age of postmenopausal women was 68.26 ± 8.31 years. The fractal dimension of four regions of interest was found to be statistically significant with a P value of less than 0.01 and GLCM characteristics, including contrast (P = 0.812), correlation (P = 0.230), energy (P = 0.215), and homogeneity (P = 0.322), did not show any significant differences. Strut traits of the four regions of interest identified notable disparities in 15 of the 19 included characteristics, In Region of Interest (ROI) 1, there was a reduction in both the overall length of the struts and the region of high density, with (p-values of 0.061 and 0.063), respectively. Additionally, attributes such as strut length and node traits were found to have increased, with a p-value of less than 0.01, which was noticed in postmenopausal women. In ROI 2 Strut length and terminal traits were increased, (p-value of <0.01) and there was a decrease in the number of nodes in strut length, periphery and node terminus (p-value of 0.114,0.198 and 0.073) respectively. ROI 3 all the strut length, nodes and terminus traits were increased, (p-value of <0.01) and periphery and total area were decreased (p-value of 0.66) ROI 4, postmenopausal women experienced a decline in periphery and total area, (p-value of 0.71) whereas there was an increase in attributes associated with strut length and node (p-value of<0.01). The mean difference of four regions of interest between the two groups was calculated and recorded for comparison (Table 1).

**Fig. 4:**
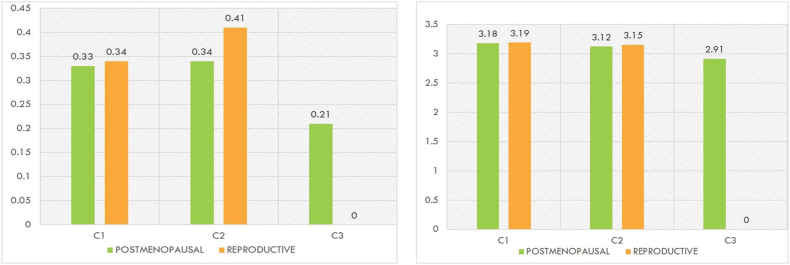
(a) Mean difference of panoramic mandibular index and mandibular cortical index among the Pre- and postmenopausal age groups, 4(b) Mean difference of mental index and mandibular cortical index among the pre- and postmenopausal age groups.

Among a group of 136 postmenopausal women, the mandibular cortical index revealed that fourteen of them belonged to the C3 type, sixty-four individuals belonged to the C2 type, and fifty-eight individuals belonged to the C1 type. The Panoramic mandibular index is decreased in postmenopausal age women with a mean difference of 0.33 in the C1 type, 0.34 in the C2 type and 0.21 in the C3 type. A statistically significant difference with (p-value of 0.035) in C1 type and (p-value of <0.01) in C2 was observed in the Mental index and (p-value of 0.043) in C1 type and (p-value of <0.01) in C2 was observed in Panoramic mandibular index in a comparative analysis between postmenopausal and reproductive-age women (Fig. 4a and b).

## 4 Discussion

Osteoporosis of the mandible can lead to a reduction in bone density, which can compromise the structural integrity of the jawbone. This can result in an increased risk of complications, including tooth loss, bone fragility, fractures, and difficulties in dental procedures such as implants and extractions. A literature search found a link between changes in the microarchitecture of the jaw bone and osteoporosis. The relationship can be observed using dual-energy X-ray absorption (DXA), Image-J, and MATLAB.^12^^,^^13^ However, these techniques are expensive when compared with digital panoramic radiographs. Orthopantomograms, though not gold standards in the diagnosis of osteoporosis, but can effectively function as an ideal, cost-effective screening tool to detect changes in bone mineral density. A digital panoramic radiograph is frequently prescribed for orofacial pathology and, hence, can serve a dual purpose in screening for osteoporosis in susceptible individuals. Several investigations have endeavoured to detect this modification in panoramic radiography, which has been employed in routine dental treatment for many years.

The posterior mandible was chosen to select the region of interest as there is no overlapping of the cervical spine, which could otherwise lead to inaccurate results. The posterior portion of the focal trough is less affected by the patient's position, resulting in a thicker and less hazy image compared to the anterior region. Ghost artefacts and variations in object thickness within the focus trough are frequently observed in panoramic radiography, leading to potential alterations in the image's grey scale and subsequent image processing outcomes. We employed density correction as a means of mitigating these significant fluctuations on a wide scale.^14^ For inter-observer reliability, twenty radiographs of the sample were randomly selected and analysed by the two observers after 1 month using Cronbach's alpha with good inferences of 0.8.

In image texture analysis, different regions of interest (ROIs) are used to study and characterise the texture properties of specific areas within an image. Texture refers to the spatial arrangement of pixel intensities in an image and plays a crucial role in various image processing and vision applications in computers, such as pattern recognition, segmentation, and object detection. Using different ROIs allows us to gain insights into specific features or variations present in different parts of the image. Hence, four regions of interest were selected in our study, according to Hwang et al.^10^

Radio-morphometric indices are specific measurements that are based on the distances and anatomical landmarks seen in an orthopantomogram. These indices are used for specific dental and skeletal assessments and are usually standardised to aid in diagnosis. It is well-established and widely used in dental and skeletal assessments. The disadvantage is that there is limited information about the overall bone microarchitecture, and the accuracy of measurements relies on the positioning and quality of the image.^15^^,^^16^ Image texture analysis is a technique used to quantify the spatial variations of pixel intensities in an image. It provides information about the patterns, roughness, and complexity of the structures present in the image. In orthopantomograms, texture analysis can be used for various purposes, such as the detection of anomalies, fracture detection, and identification of bone quality like the trabecular bone pattern and density, which are relevant for evaluating bone health and osteoporosis risk.^17^

Image texture analysis and radio-morphometric indices are two different approaches used in the analysis of trabecular bone patterns. Image texture analysis requires advanced image processing techniques and algorithms, and the interpretation of results may be challenging without proper training.^18^ Whereas radio morphometric indices have standardised measurements with established reference values for comparison,^18^^,^^19^ they are relatively easy to apply and interpret, even by non-specialists.

The fractal dimension has been reported to be useful in detecting osteoporosis in panoramic radiography, whereas other studies have reported different results.^19^^,^^20^ Our study found statistically significant differences in fractal dimension and statistically insignificant differences in GLCM features, similar to Kavitha et al.^12^ study, but we are unable to compare their results to ours since there were differences in the region of interest (ROI) and the number of grey-level co-occurrence matrix (GLCM) variables. In line with the Hwang et al.^10^ study, strut features revealed statistically significant differences between pre-and postmenopausal age.

The radio-morphometric indices used in OPG, such as the mandibular cortical width, the mandibular cortical index, the mental index, and the panoramic mandibular index gonial index, are chosen based on the clinical question and the bone health aspect being evaluated. Each index provides unique information about bone structure and density. It can be calculated objectively, reducing the potential for subjective interpretation or bias. Researchers often use a combination of these indices to gain a comprehensive understanding of the patient's bone health and to make informed treatment decisions.

The mandibular cortex has mostly been evaluated for the identification of osteoporosis with the use of panoramic radiography (mandibular cortical index). Even so, the MCI is limited in its ability to be fully replicated as it does not include any measurements or calculations, instead relying on visual evaluations.^21^, ^22^ In the present study, fourteen out of 136 patients showed the C3 type of MCI among postmenopausal women. Gulsahi et al. conducted a study in patients with the C3 type of MCI, where high-risk individuals were considered for osteoporosis.^,^ Kiswanjaya et al.,^23^ Hastar et al.^24^ and Dagestan et al.^25^ determined that MCI can serve as a supplementary method for evaluating reduced skeletal bone mass. and PMI is considered one of the most accurate radio-morphometric indices. According to the findings of Dagestan S. et al.,^25^ the PMI in the present study decreased in postmenopausal women. The comparison between MI and PMI among postmenopausal and reproductive-age women was statistically significant in this study.

Hence, the findings of this study suggest that fractal analysis can capture subtle changes in the structural complexity of bones, allowing for the detection of early signs of osteoporosis that might not be apparent through traditional methods. GLCM serves as a supplementary tool for analyzing the spatial arrangement of intensity values and their interconnections. This is essential for characterizing different textures present in an image. In strut analysis, which assesses bone strut distribution and organization, reveals the bone's biomechanical qualities, including its strength and fracture susceptibility. While these advanced image processing techniques offer several advantages over traditional radio-morphometric indices, their implementation may require specialized expertise and computational resources. Integrating these methods with conventional techniques can enhance the accuracy and reliability of osteoporosis detection in OPG images.

The limitations are that the sample size used in the current study was considerably smaller and only fundamental medical records and radiographic subsets were archived, whereas demographic information and hormonal state were not accessible. Studies with larger samples are to be conducted at the multicentric level to validate the results of our study. There was no incorporation of technology with algorithm development and interdisciplinary collaboration, which could have made the entire process more feasible and less time-consuming. These areas have the potential to significantly impact various healthcare modalities by providing valuable insights and aiding decision-making processes.

## 5 Conclusion

In the recent trend of evidence-based dentistry, digital panoramic radiography is routinely used as a cost-effective, patient-compliant, screening, and patient-education tool. It is a known fact that osteoporosis is common among women, especially at postmenopausal age. Hence, panoramic radiographs that have been taken for dental treatment can be used as an affordable screening standard for the diagnosis of osteoporosis in women. Future research incorporating artificial intelligence can revolutionize image analysis and support clinical decision-making.

## Conflicts of interest

No conflict of interest.

## Contributions

All the co-authors listed in the manuscript have made substantial contributions to the research and preparation of the manuscript and have reviewed and approved the final version of the paper.

## Abbreviations

OPG: Orthopantomogram
ROI: Region of interest
GLCM: Grey level co-occurrence matrix
MCI: Mandibular cortical index
PMI: Panoromic mandibular index
MI: Mental index
